# Supplementary material for: Ownership and Agency of an Independent Supernumerary Hand Induced by an Imitation Brain-Computer Interface
Source: PLoS One. 2016 Jun 15;11(6):e0156591. doi: 10.1371/journal.pone.0156591 (PMC4909224; doi:10.1371/journal.pone.0156591)
Supplement: S2 File — Document describing the data provided in S1 File. (PDF) [file pone.0156591.s002.pdf]

Manuscript Data Variables Explained

IBCI.GSR = (threats(1=pre, 2=early, 3=late, 4=post), hands(1=L, 2=R), subjects)
IBCI.Questionnaire = (subjects, questions)
IBCI.ControlQuestionnaire = (subjects, questions)
IBCI.Temperature = (time, subjects)

RHI.Questionnaire = (subjects, questions)
RHI.ControlQuestionnaire = (subjects, questions)
RHI.Temperature = (time, subjects)

RTHI.Questionnaire = (subjects, questions)
RTHI.ControlQuestionnaire = (subjects, questions)
RTHI.Temperature = (time, subjects)

In questionnaire variables, the questions are ordered as shown in the manuscript. The 7 step visual analogue scale (---,--,-,0,+,++,+++) corresponds to (1,2,3,4,5,6,7) in the data respectively.

In the Temperature variables; temperature is given in °C, the time dimension is in seconds and aligned at illusion onset.
